# Letter in response to the Article published by Reich O, Rapold R, Flatscher-Thöni M. An empirical investigation of the efficiency effects of integrated care models in Switzerland. Int J Integr Care 2012,12:1–12

**DOI:** 10.5334/ijic.841

**Published:** 2012-03-06

**Authors:** Eva Blozik, Jan von Overbeck

**Affiliations:** Swiss Center for Telemedicine Medgate, Basel, Switzerland; Swiss Center for Telemedicine Medgate, Basel, Switzerland

Dear Editor,

The paper by Reich et al. recently published in the IJIC compares healthcare expenditure of patients insured in a contracted capitated model, a contracted non-capitated model, and a telemedicine model [1]. Their evaluation showed that the telemedicine model was associated with an efficiency gain of 3.7% as opposed to 21.2% for the contracted capitated and 15.5% for the non-capitated model. The analyses were restricted to persons who claimed reimbursement for health care expenses during the study period. As the authors themselves state, this restriction is a potential source of bias, which we believe has major implications for the interpretation of the study results.

Telemedicine models usually include teleconsultation (accurate history taking and counselling by trained medical personnel) and teletriage (allocation of the appropriate point of care and time to treat). The objective of such models is to avoid unnecessary face-to-face consultations generated by health problems that may be solved over the phone. Patients are assisted through information, empowerment, recommendations for selfcare measures and teleprescription [2].

In Switzerland, teleconsultation and triage services are billed in bulk to the insurers. The fees are not linked to individual patients, and therefore the teleconsultation itself does not result in expenditure for those individuals. If they are not referred to a face-to-face consultation, they incur no expenses at all, except possibly the costs of medication. Restricting the analysis to those persons claiming reimbursement for health care expenses therefore excludes those whose health problem is successfully addressed using selfcare measures.

Further, as pointed out by the authors, many relatively healthy persons in Switzerland have a high deductible, and often do not submit bills for reimbursement when they have been treated for simple or short ailments, or have had to spend modest amounts on medication. This also tends to skew the analysis towards less healthy persons, who are more likely to be referred to a face-to-face consultation by their telemedical physician and to incur higher costs. We believe that this selection bias substantially reduces the measured effect of the telemedicine model.

Additionally, telemedicine models differ: the most basic services are based purely on triage by nurses or other general healthcare personnel. Other more advanced services provide triage and teleconsultation by physicians trained in telemedicine [3]. Here, the options of teleprescription and telediagnostics increase the number of situations in which selfcare is appropriate [4]. Furthermore, there are different levels of compliance the patients are obliged to meet when receiving a telemedical recommendation. Some models include financial incentives for compliance. The data reported on in the study by Reich et al. is based on a telemedicine model provided by non-physician personnel with a very low level of obligation for compliance. We are convinced that a similar analysis of a physician-based model would reveal a considerably higher effect.
